# Matrine Inhibits CNS Autoimmunity Through an IFN-β-Dependent Mechanism

**DOI:** 10.3389/fimmu.2020.569530

**Published:** 2020-09-25

**Authors:** Yao-Juan Chu, Wen-Di Ma, Rodolfo Thome, Jie-Dan Ping, Fang-Zhou Liu, Meng-Ru Wang, Ming-Liang Zhang, Guangxian Zhang, Lin Zhu

**Affiliations:** ^1^Department of Pharmacy, The First Affiliated Hospital of Zhengzhou University, Zhengzhou, China; ^2^Department of Neurology, Thomas Jefferson University, Philadelphia, PA, United States; ^3^Department of Clinical Laboratory, Key Clinical Laboratory of Henan Province, The First Affiliated Hospital of Zhengzhou University, Zhengzhou, China; ^4^Henan Province Chinese Medicine Research Institute, Zhengzhou, China; ^5^Henan Province Engineering Laboratory for Clinical Evaluation Technology of Chinese Medicine, The First Affiliated Hospital of Henan University of Chinese Medicine, Zhengzhou, China

**Keywords:** matrine, multiple sclerosis, experimental autoimmune encephalomyelitis, IFN-β, IL-10, IL-27

## Abstract

Matrine (MAT), a quinolizidine alkaloid component derived from the root of *Sophora flavescens*, suppresses experimental autoimmune encephalomyelitis (EAE), the animal model of multiple sclerosis (MS), by inducing the production of immunomodulatory molecules, e.g., IL-10. In an effort to find the upstream pathway(s) of the mechanism underlying these effects, we have tested certain upregulated immunomodulatory molecules. Among them, we found increased levels of IL-27 and IFN-β, one of the first-line MS therapies. Indeed, while low levels of IFN-β production in sera and type I interferon receptor (IFNAR1) expression in spinal cord of saline-treated control EAE mice were detected, they were significantly increased after MAT treatment. Increased numbers of CD11b^+^IFN-β^+^ microglia/infiltrating macrophages were observed in the CNS of MAT-treated mice. The key role of IFN-β induction in the suppressive effect of MAT on EAE was further verified by administration of anti-IFN-β neutralizing antibody, which largely reversed the therapeutic effect of MAT. Further, we found that, while MAT treatment induced production of IL-27 and IL-10 by CNS microglia/macrophages, this effect was significantly reduced by IFN-β neutralizing antibody. Finally, the role of IFN-β in MAT-induced IL-27 and IL-10 production was further confirmed in human monocytes *in vitro*. Together, our study demonstrates that MAT exerts its therapeutic effect in EAE through an IFN-β/IL-27/IL-10 pathway, and is likely a novel, safe, low-cost, and effective therapy as an alternative to exogenous IFN-β for MS.

## Introduction

Multiple sclerosis (MS) and its animal model, experimental autoimmune encephalomyelitis (EAE), are chronic inflammatory demyelinating diseases of the central nervous system (CNS) (1, 2). The pathological features of MS are immune system imbalance, CNS inflammation, demyelination and neuro-axonal damage (3–6). Myelin-reactive Th1 and Th17 cells are activated in the periphery and infiltrate into the CNS, along with B cells, macrophages, dendritic cells and neutrophils. In the CNS, these cells produce proinflammatory cytokines IFN-γ, IL-17, GM-CSF, and IL-23, activate microglia, and promote cell- and antibody-mediated immunity. In contrast, immunoregulatory cytokines, such as IL-4, IL-10, and IL-27, may be protective (1, 2). The effectiveness of current therapies is limited (7, 8). Novel therapies for MS that are more effective, and with fewer side effects than those currently in use are, thus, crucially required.

Matrine (MAT), a quinolizidine alkaloid component extracted from the root of *Sophora flavescens*, has been shown to have anti-viral, immunoregulatory, anti-inflammatory, anti-allergic and anti-tumor effects (9). We have previously reported that MAT decreased clinical EAE severity, reduced CNS inflammatory infiltration and demyelination, inhibited production of inflammatory cytokines/chemokines, and promoted the production of anti-inflammatory molecules such as IL-4 and IL-10 (10). In addition, MAT promoted regeneration of injured myelin sheaths by inhibiting the pathway of nerve regeneration induced by Nogo-A (11). These results suggested that MAT had significant anti-inflammatory potential for the treatment of EAE/MS. However, the mechanisms underlying these effects are still unknown.

To define the mechanism underlying MAT action in EAE suppression, we tested certain known upstream signaling pathways that regulate IL-10 expression in the CNS and splenocytes of MAT-treated EAE mice. Among those molecules upregulated, IFN-β was of high interest, given its effect in IL-10 induction (12–14) and as a first-line therapy for MS (7, 8). IFN-β suppresses EAE, and its induction is beneficial in treatment of autoimmune neuroinflammation (15). The key role of IFN-β induction in the effect of MAT treatment in EAE was further confirmed by neutralizing antibody *in vivo* and in human monocytes *in vitro*.

## Materials and Methods

### Animals, EAE Induction and Evaluation

Female C57BL/6 mice, 8–10 weeks old, were purchased from the Beijing HFK Bioscience Co., Ltd., (Beijing, China), and bred in specific pathogen-free conditions at the Henan Province Traditional Chinese Medicine Research Institute, China. All experimental procedures and protocols were approved by the Bioethics Committee of Zhengzhou University and followed the institutional guidelines and regulations. Every effort was made to reduce animal suffering.

EAE was induced as described previously (16). Myelin oligodendrocyte glycoprotein 35–55 (MOG35-55) peptide lyophilized powder (Invitrogen, California state, United States) was diluted to 3 mg/ml with 0.1 M PBS, emulsified with the same volume of complete Freud’s adjuvant (CFA) (Sigma, St. Louis, MO, United States) containing 4 mg/ml heat-killed mycobacterium tuberculosis H37RA (Becton, Dickinson and Company, NJ, United States). The mice were injected subcutaneously at four separate points on the back with 200 μl of antigen emulsion. In addition, mice were injected intraperitoneally (i.p.) with 200 ng/100 μl of pertussis toxin (Sigma-Aldrich, Brøndby, Denmark) at the time of immunization and 48 h later. Clinical signs of EAE were scored daily by two researchers in a blind fashion as follows: 0 = no clinical score, 1 = loss of tail tone, 2 = hind limb weakness, 3 = hind limb paralysis, 4 = forelimb paralysis, 5 = moribund or death. A cumulative clinical score was calculated for each mouse by adding the daily scores from the day of onset until the end of treatment.

### MAT and Neutralizing IFN-β Monoclonal Antibody (mAb) Treatment

Immunized mice were randomly divided into two groups (*n* = 10 each group): (1) Saline: Immunized mice received 100 μl normal saline per day i.p. from day 7 post immunization (p.i.). (2) MAT: Immunized mice were injected i.p. with 150 mg/kg (20 ml/kg) in 100 μl normal saline per day MAT (Jiangsu Chia-tai Tianqing Pharmaceutical Co., Jiangsu, China) from day 7 p.i.

The role of IFN-β in MAT’s effect was further studied by neutralizing this cytokine with monoclonal antibodies. Briefly, immunized mice were randomly divided into four groups (*n* = 10 each group). (1) MAT alone: Immunized mice received 150 mg/kg (20 ml/kg) in 100 μl normal saline per day i.p. injection of MAT, starting from day 7 p.i. (2) Anti-IFN-β alone: Immunized mice received 250 μg/kg (10 ml/kg) neutralizing IFN-β mAb in 100 μl normal saline (Abcam, Cambridge, United Kingdom) on days 12 and 14 p.i. (at EAE onset). (3) MAT + anti-IFN-β: Immunized mice received IFN-β mAb and MAT as described in Groups 1 and 2 above. (4) Saline alone: Immunized mice that received 100 μl normal saline i.p. per day from day 7 p.i. served as untreated control.

### Isolation of Human Monocytes, MAT Treatment and Cytokine Production

Blood samples were collected from healthy individuals and the peripheral blood mononuclear cells (PBMCs) were enriched after centrifugation in Ficoll gradient. CD14^+^ monocytes were positively isolated using microbeads following the manufacturer’s instructions (Miltenyi Biotec). One million cells were seeded in 24 well plates in Iscove’s Modified Dulbecco Medium (IMDM) supplemented with 10% Fetal Bovine Serum, 1X Glutamine/Streptomycin/Penicillin (Gibco) and 1X β-mercaptoethanol (Gibco). To induce activation of monocytes, cells were treated with 500 ng/ml of lipopolysaccharide (LPS, Sigma-Aldrich) at 37°C in the presence or absence of MAT (100 μM). A total of 18 h later, supernatants were collected and kept at −20°C until used in ELISA assay.

### Histopathological Evaluation

On day 19 p.i., mice were sacrificed and perfused with normal saline. Lumbar enlargements of the spinal cords were quickly removed, fixed with 4% paraformaldehyde, neutral-buffered formalin, and embedded in paraffin. Inflammatory infiltration was determined by hematoxylin and eosin (H&E) staining and demyelination by Luxol fast blue (LFB) staining. Histopathological examination was performed and scored in a blinded fashion as follows (17): For inflammation: 0, no inflammatory cells; 1, a few scattered inflammatory cells; 2, organization of inflammatory infiltrates around blood vessels; 3, extensive perivascular cuffing with extension into parenchyma. For demyelination: 0, none; 1, rare foci; 2, a few areas of demyelination; and 3, large (confluent) areas of demyelination. Scores of demyelination and inflammation were calculated by Image-Pro Plus 6.0 software. For each mouse, three histological sections were analyzed and their average scores were calculated.

### Double-Labeling Immunofluorescence Assay

Lumbar spinal cords were immediately harvested after extensive perfusion on day 19 p.i., fixed with 4% paraformaldehyde, and cut into 5-μm-slices for immunofluorescence assay. Briefly, non-specific binding was blocked with 3% bovine serum albumin (BSA) (Serotec, United Kingdom) and permeated with 0.3% Triton X-100 in 1% BSA-PBS for 30 min. Slides were then incubated with primary antibodies-mouse anti-CD4, mouse anti-CD11b (both IgG; Proteintech, Wuhan, China), rabbit anti-IFN-β, rabbit anti-IL-10 and rabbit anti-IL-27 (all IgG; Abcam, Cambridge, United Kingdom) in blocking solution overnight at 4°C, followed by incubation with corresponding secondary antibodies-goat anti-rabbit Cy3 conjugate (IgG; Proteintech, Wuhan, China) and donkey anti-mouse Alexa Fluor 488 (IgG; Jackson ImmunoResearch, PA, United States) for 2 h at room temperature. After three additional washes with PBS, samples were counterstained with DAPI for 4′,6-diamidino-2-phenylindole (DAPI, Roche, Basel, Switzerland), washed with PBS and mounted. Images were captured by confocal microscope (Olympus FluoView FV1000).

### ELISA

Sera were harvested on day 19 p.i. when mice were sacrificed. Supernatants from human monocyte cultures were harvested after 18 h. of culture and kept at −20°C until use. IFN-β, IL-10, IL-27, and TNF-α concentrations were measured by ELISA following the manufacturer’s instructions (R&D Systems, United States). Samples were quantified by comparison with the standard curves. Determinations were performed in duplicate in ten samples for each group. Results were analyzed by GraphPad Prism 6 software.

### qRT-PCR

Total RNA was extracted from the spinal cord tissues using Trizol reagent (Transgene Biotech Co., Beijing, China) following the manufacturer’s instructions. Complementary DNA (cDNA) synthesis was performed using Reverse Transcription Kit (Thermo Fisher Scientific, MA, United States). Relative quantification of target gene expression was determined by ABI Prism^®^ 7500 Sequence Detection System (Biosystems, CA, United States). Primer sequences used for IFNAR1 are listed as follows: Forward 5′-TCCCCGCAGTATTGATGAGT-3′, Reverse 5′-CTGGTCTGTGAGCTGTACTT-3′.

### Statistical Analysis

GraphPad Prism8.0 (GraphPad Software, Inc., La Jolla, CA, United States) was used for statistical analysis. All values are presented as mean ± SD. Comparisons between groups were analyzed using a two-tailed Student’s *t*-test, one-way ANOVA analysis of variance followed by Bonferroni correction for measurement data, or Mann–Whitney U test for ordinal data, as appropriate. *P* values less than 0.05 were considered statistically significant.

## Results

### MAT Alleviated Clinical Severity of EAE Mice

**FIGURE 1:**
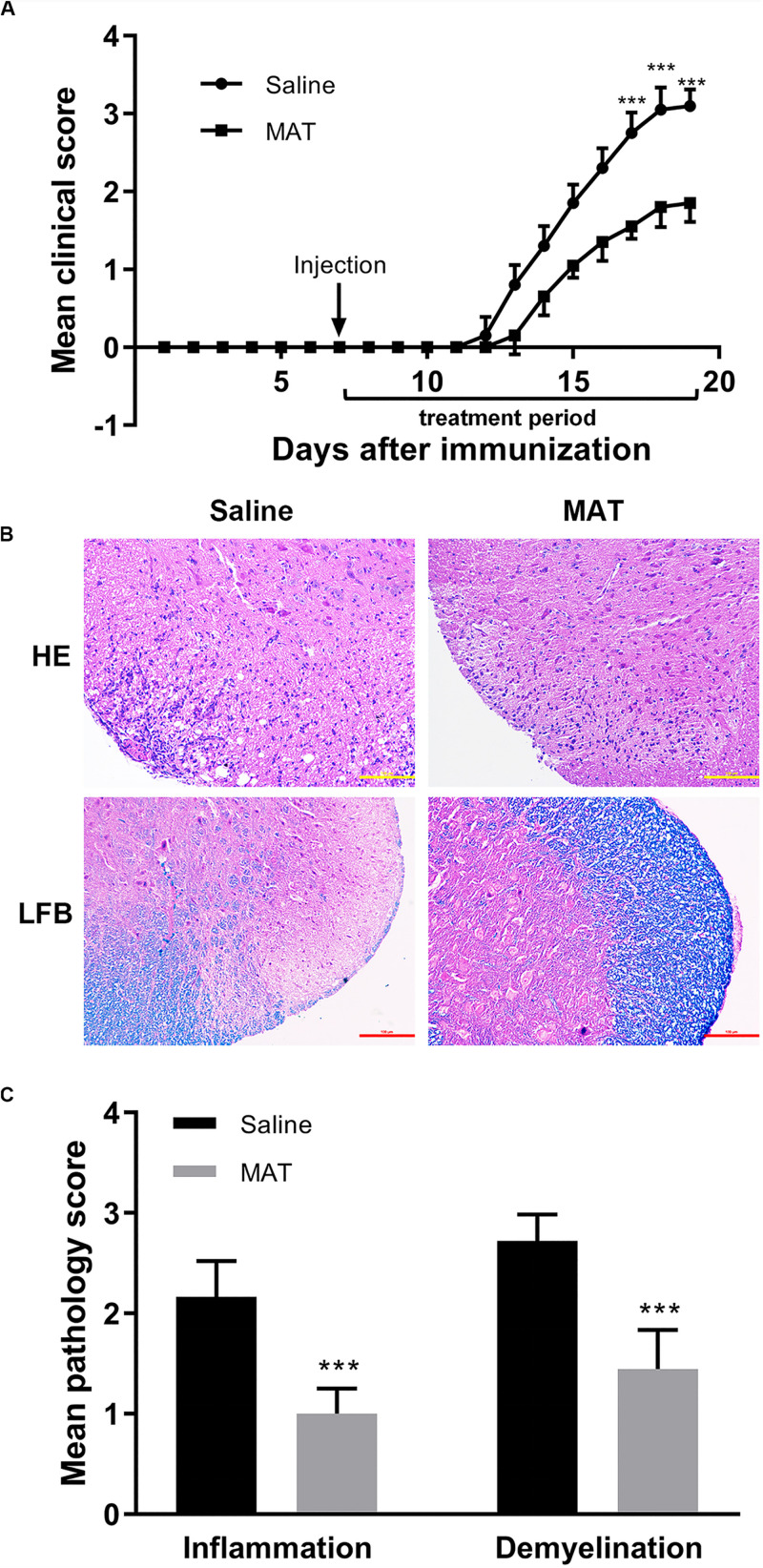
MAT ameliorated the severity of EAE. EAE was induced in female C57BL/6 mice by MOG35-55 + CFA. Mice received MAT treatment (150 mg/kg in 100 μl normal saline per day, i.p. injection) from day 7 to day 19 p.i., and mice that received the same volume of saline served as control. **(A)** Clinical score was monitored as described in the “Materials and Methods” section. Data represent mean clinical score ± SD (*n* = 10 mice per group). Mice were sacrificed on day 19 p.i., and spinal cords were harvested after extensive perfusion. **(B)** H&E staining was performed for detection of inflammation and Luxol fast blue (FLB) staining for demyelination. **(C)** Mean score of inflammation and demyelination. Data represent mean ± SD (*n* = 10 mice per group). ***P < 0.001.

Saline-treated immunized mice showed the first signs of EAE on day 12 p.i., while the MAT-treated group did so on day 13 p.i. Mean clinical scores were significantly reduced in the MAT-treated group compared to the saline-treated group (Figure 1A). In order to assess EAE neuropathology, the lumbar enlargement of spinal cords was assayed for H&E and LFB staining. Extensive inflammatory infiltration was found in the white matter of spinal cords of saline-treated EAE mice, and this infiltration was significantly inhibited by MAT treatment (Figure 1B). Consistent with inflammation, severe demyelination was also observed in the spinal cords of the saline-treated group, and this was significantly decreased in the MAT-treated group (Figure 1C).

### MAT Induced IFN-β Production in Periphery and the CNS

**FIGURE 2:**
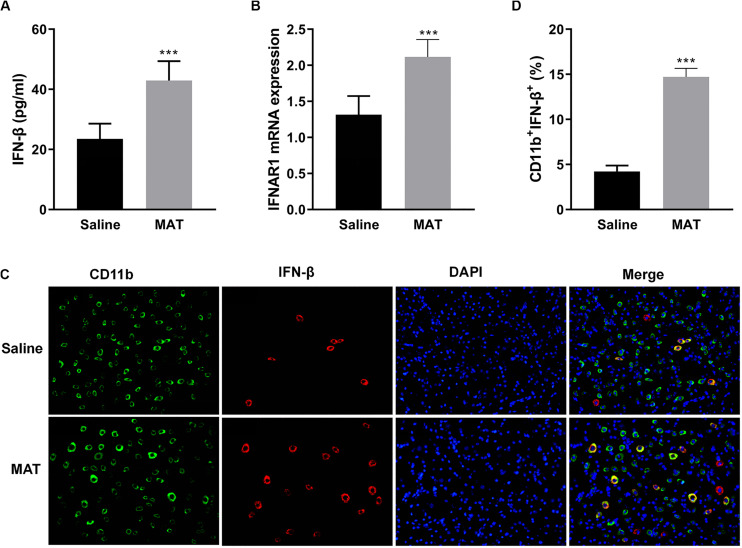
MAT-induced IFN-β levels in serum and in the CNS. EAE mice described in Figure 1 were sacrificed on day 19 p.i. Sera and spinal cords from each individual mouse were harvested. **(A)** Serum IFN-β concentrations were measured by ELISA. **(B)** mRNA expression of IFNAR1 in the spinal cords was measured by qRT-PCR. **(C)** IFN-β secretion of CD11b^+^ cells (microglia and infiltrating macrophages) in the spinal cords was determined by immunofluorescence double staining. **(D)** Quantitative analysis of percentage of IFN-β^+^ cells among total CD11b^+^ cells. Values represent mean ± SD (*n* = 10 mice per group). ***P < 0.001.

Our previous studies had shown that MAT reduced the severity of EAE by significantly increasing serum production of IL-10, a potent immunomodulatory cytokine (10). To define the mechanism of MAT inducing IL-10, we examined the production of IFN-β, one of the upstream molecules of IL-10 expression (18). As shown in Figure 2A, while serum of saline-treated EAE mice contained a low level of IFN-β production, this production was significantly increased in the serum of MAT-treated EAE mice. Further, MAT treatment significantly upregulated the expression of type I interferon receptor (IFNAR1) in spinal cord of EAE mice compared to saline-treated control EAE mice (Figure 2B), indicating an enhanced IFN-β responsiveness of CNS cells.

To evaluate IFN-β expression in CNS inflamed foci after MAT treatment, double-labeling immunofluorescence essay was performed for IFN-β and CD11b^+^ cells, i.e., microglia and infiltrating macrophages, given that this cell lineage is the main source of IFN-β. As shown in Figures 2C,D, only a few CD11b^+^IFN-β^+^ cells were observed in saline-treated EAE mice, while there were greater numbers of these cells in MAT-treated mice. The difference between these two groups was statistically significant. We thus postulated that MAT could induce CD11b^+^ cells to produce IFN-β.

### The Therapeutic Effect of MAT Was Diminished by Neutralizing IFN-β mAb

**FIGURE 3:**
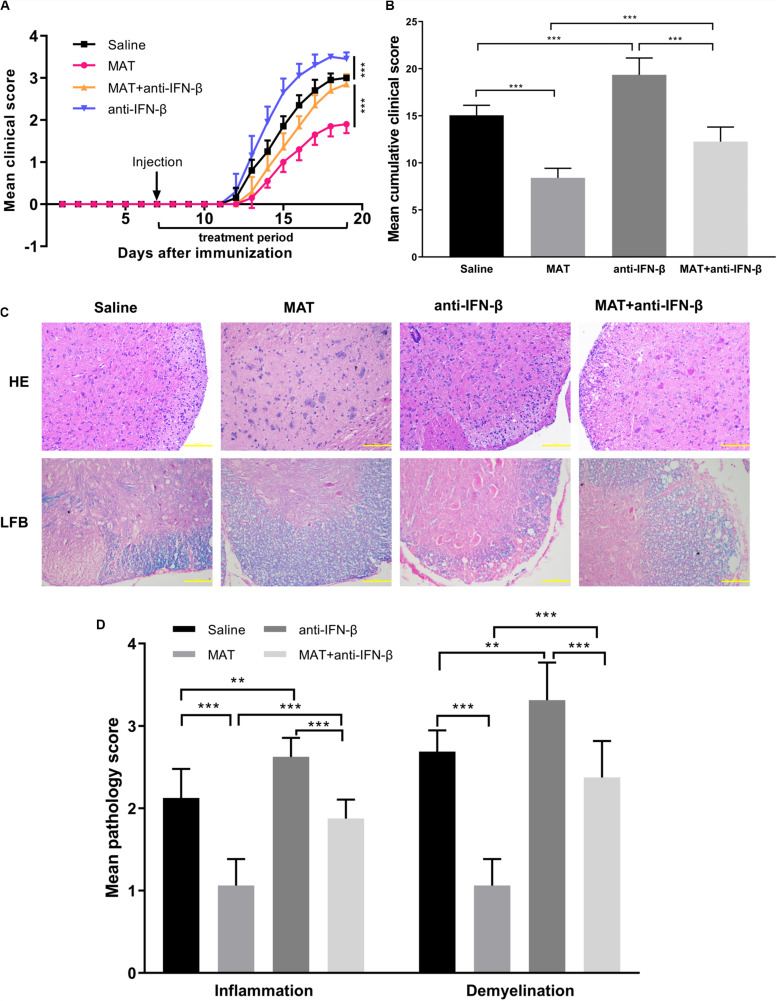
The effect of MAT was diminished by neutralizing anti-IFN-β mAb. EAE was induced in female C57BL/6 mice by MOG35-55, and received MAT and/or neutralizing anti-IFN-β mAb as described in the “Materials and Methods” section. Clinical score was monitored daily. **(A)** The mean clinical scores. Data represent mean clinical score ± SD (*n* = 10 mice per group). **(B)** Mean cumulative clinical score obtained by adding the daily scores from the day of onset until the end of treatment for each mouse. Data represent cumulative clinical score ± SD (*n* = 10 mice per group). Mice were sacrificed on day 19 p.i., and spinal cords were harvested after extensive perfusion. **(C)** H&E staining was performed for detection of inflammation and Luxol fast blue (FLB) staining for demyelination. **(D)** Mean score of inflammation and demyelination. Data represent mean ± SD (*n* = 10 mice per group). **P < 0.01, ***P < 0.001.

To further verify the role of IFN-β production in the effect of MAT treatment, neutralizing IFN-β mAb was used in MAT-treated mice. Compared to the saline-treated group, the anti-IFN-β group developed a more severe progressive disease course, indicating a role of endogenous IFN-β production in EAE mice, though undetectable, as a negative feedback to control CNS inflammation. While the mean clinical score and cumulative clinical score were significantly reduced in the MAT-treated group, this effect was significantly diminished after IFN-β blockade, indicating that IFN-β induction plays a key role in MAT treatment (Figures 3A,B). Consistent with clinical score, HE staining showed an extensive inflammatory infiltration in the spinal cords of EAE mice, while this infiltration was significantly enhanced by anti-IFN-β and inhibited by MAT treatment. Furthermore, MAT treatment was able to significantly reduce demyelination in the CNS of EAE mice, compared to the MAT + anti-IFN-β group (Figures 3C,D). Thus, these observations indicate that IFN-β induction plays a key role in the effect of MAT in EAE.

### IFN-β Concentrations in the Periphery and the CNS After MAT/Anti-IFN-β Treatment

**FIGURE 4:**
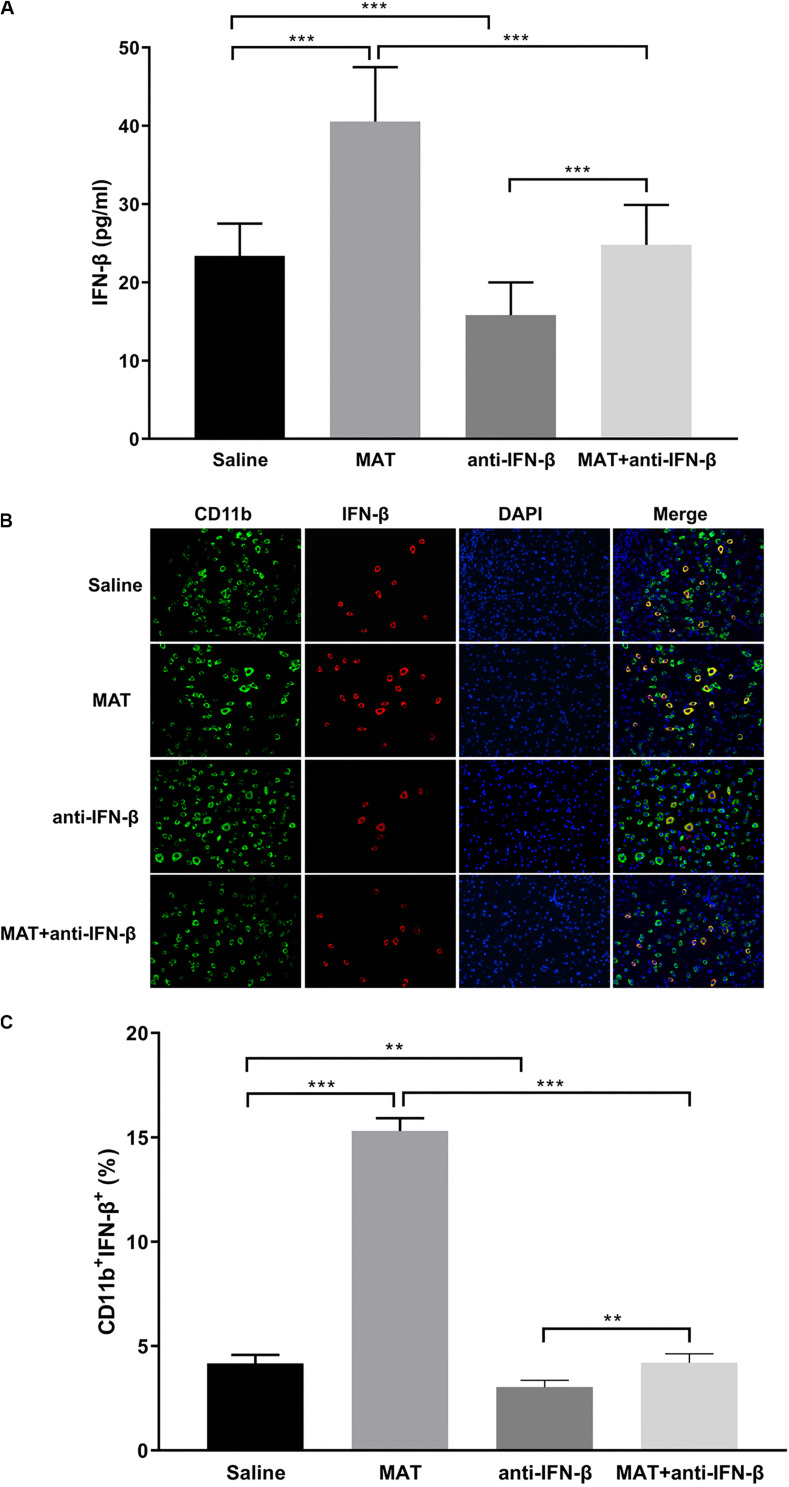
IFN-β levels in serum and in the CNS after MAT and/or anti-IFN-β mAb. Sera and spinal cord tissues from each individual mouse were harvested on day 19 p.i. **(A)** Concentrations of IFN-β in the sera were measured by ELISA. **(B)** IFN-β secretion of CD11b^+^ cells (microglia and infiltrating macrophages) in the spinal cords was determined by immunofluorescence double staining. **(C)** Quantitative analysis of percentage of IFN-β^+^ cells among total CD11b^+^ cells. Data represent mean ± SD (*n* = 10 mice per group). **P < 0.01, ***P < 0.001.

To confirm the effect of anti-IFN-β in MAT treatment, serum concentrations of IFN-β were determined by ELISA, and spinal cord tissues were examined by double-labeling immunofluorescence essay. IFN-β levels in the sera were significantly increased in MAT-treated mice compared to the MAT + anti-IFN-β group, while IFN-β protein expression was lowest in the anti-IFN-β group of mice. No significant difference was found in the IFN-β level between sera of the saline-treated group and MAT + anti-IFN-β group of mice (Figure 4A).

We then evaluated the effect of anti-IFN-β on IFN-β expression in CNS inflamed foci after MAT treatment. Double-labeling immunofluorescence essay was performed for IFN-β and CD11b^+^ cells. As shown in Figures 4B,C, only a few CD11b^+^IFN-β^+^ cells were observed in EAE mice treated with saline or anti-IFN-β alone, while they were observed in a larger number in MAT-treated mice. However, the numbers of CD11b^+^IFN-β^+^ cells in the MAT + anti-IFN-β group were significantly reduced compared to those in mice treated with MAT alone. These data indicate that after neutralizing anti-IFN-β antibody blocked the immunomodulatory effect of MAT on macrophages/microglia, which produce very little IFN-β.

### MAT Increased the IL-27 and IL-10 Expression

**FIGURE 5:**
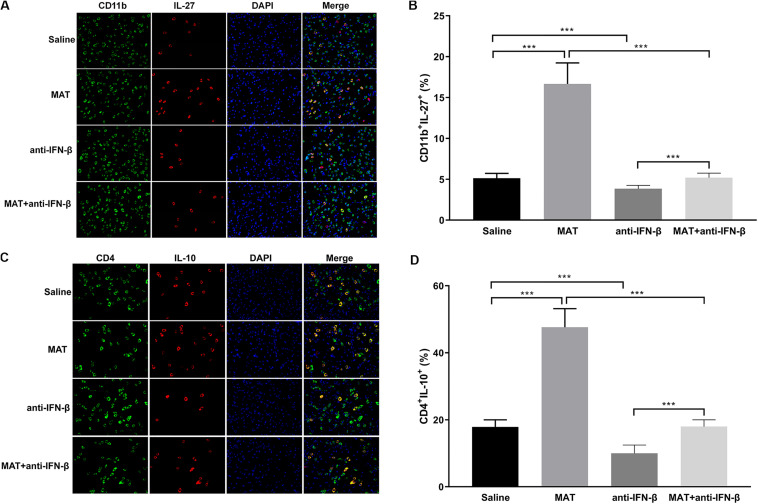
MAT increased IL-27 and IL-10 expression. Spinal cord tissues from each individual mouse were harvested on day 19 p.i. IL-27 **(A)** secretion of CD11b^+^ cells and IL-10 **(C)** secretion of CD4^+^ cells in the spinal cords was determined by immunofluorescence double staining. Quantitative analysis of percentages of IL-27 **(B)** producing cells among total CD11b^+^ cells and IL-10 **(D)** producing cells among total CD4^+^ cells. Data represents mean ± SD (*n* = 10 mice per group). ***P < 0.001.

As IL-27 and IL-10 are the downstream molecules of IFN-β, we determined the expression of IL-27 on CD11b^+^ cells and IL-10 on CD4^+^ cells by double-labeling immunofluorescence assay. We found that only a few CD11b^+^IL-27^+^ cells were observed in EAE mice treated with saline or anti-IFN-β alone, while they were observed in a larger number in MAT-treated mice. However, the numbers of CD11b^+^IL-27^+^ cells in the MAT + anti-IFN-β group were significantly reduced compared to mice treated with MAT alone (Figures 5A,B). Similarly, while the largest proportion of CD4^+^IL-10^+^ T cells was observed in mice treated with MAT alone, this was significantly reduced in the MAT + anti-IFN-β group. In contrast, only small proportions of CD4^+^IL-10^+^ T cells were observed in EAE mice treated with saline alone or anti-IFN-β alone (Figures 5C,D).

### MAT Induces IFN-β Production by Human Monocytes

**FIGURE 6:**
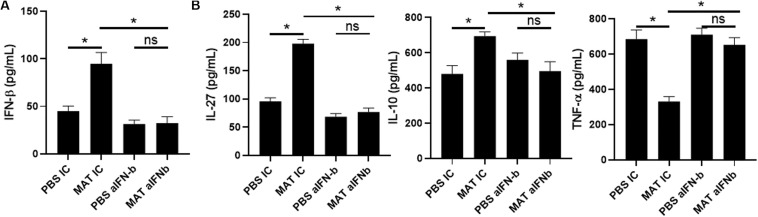
MAT induced IFN-β in human monocytes. CD14^+^ monocytes were isolated from PBMCs of healthy donors and activated for 18 h with LPS (500 ng/ml) in the presence or absence of Matrine (100 mM) and anti-IFN-β antibodies (20 μg/ml) or isotype control (IC). After 18 h of incubation period, supernatants were harvested for production of IFN-β **(A)**, IL-27, IL-10 and TNF-α **(B)**. Bar graphs represent the mean ± SEM. Representative data from one out of three independent experiments. *P < 0.05.

Our results showed that MAT induces IFN-β and IL-27 in microglia and macrophages in the CNS of EAE mice. This is likely a pathway by which MAT suppresses the clinical development of EAE. However, from the clinical perspective, it would be necessary to demonstrate that MAT can induce IFN-β in human cells. To address this question, we isolated peripheral blood CD14^+^ monocytes and treated them with LPS (500 ng/ml) in the presence or absence of MAT (100 μM) for 18 h. We found that MAT-treated monocytes had a significant increase in IFN-β production compared to controls (Figure 6A). Moreover, MAT induced IL-27 and IL-10 while suppressing TNF-α (Figure 6B). These effects were dependent on IFN-β as anti-INF-β treatment abrogated IL-27 and IL-10 production and increased TNF-α in MAT-treated cultures (Figures 6A,B). Collectively, our data show that MAT stimulates IFN-β production by microglia/monocytes in mice and in human monocytes.

## Discussion

In the present study we demonstrate that MAT induced IFN-β production, which promoted production IL-27 and IL-10, and is thus a mechanism of the immunomodulatory effects of MAT in suppressing the development of EAE. While this effect *in vivo* may represent an indirect effect through its immunomodulatory network, an *in vitro* study further confirmed a direct effect of MAT on IFN-β induction.

IFN-β, a monomeric type I IFN with both antiviral and potent anti-inflammatory effects, is also a key inducer of IL-10 and a suppressor of Th17 differentiation (19). IFN-β signaling is mediated through the common type I IFN receptor and proceeds through the classic JAK/STAT signaling pathway (20). IFN-β inhibits CCL2/CCL5 induced-T cell migration by inhibiting p38-MAPK and ERK1/2 activation and reduces the expression of chemokines and chemokine receptors expressed by encephalitogenic Th1/Th17 cells, thereby decreasing their migration into the CNS (21). It was found that sera of relapsing remitting multiple sclerosis (RRMS) patients have a deficient endogenous IFN-β production, as determined by an IFN-β–reactive cell line. Endogenous IFN-β suppresses the basal Th17 cytokine secretion of RRMS patients, and blockade of its downstream pathway significantly diminished this effect (22). A deficiency in secretion of endogenous IFN-β might therefore contribute to the reduction of the Th17-mediated autoimmune response in RRMS patients (22).

While long-term administration of high-dose recombinant IFN-β has been a first-line MS therapy, an ideal approach would have the capacity to enhance endogenous IFN-β production. Indeed, several approaches have been reported to induce endogenous IFN-β. For example, self-assembling peptides, after being formed as immunomodulatory amyloid fibrils, effectively suppressed EAE, with induction of a significant amount of type 1 IFN (23). IFN-β production can also be induced by certain natural compounds. Berberine, a purified compound of the crude extract of an anti-inflammatory herb coptis, remarkably upregulated IFN-β expression in a breast cancer cell line, thus demonstrating potential as a promising adjuvant for breast cancer treatment (24). Similar results were also observed in an extract of Huanglian (Coptidis rhizome), a widely used herb in traditional Chinese medicine (25). While production of IFN-β and IL-10 was significantly decreased in the colonic mucosa of ulcerative colitis rats, their production was significantly upregulated upon herb-partitioned moxibustion (26). Fractions obtained from the aqueous extract of *B. coccineus*, a plant commonly found in west and tropical Africa, significantly stimulated IFN-β expression in PBMCs, causing a seven-fold increase of its transcript (27). More recently, the Bowman-Birkman Inhibitor (BBI), a soybean derivative, was shown to induce IFN-β production, IFNAR1 gene expression, and their downstream signaling molecules, as a mechanism underlying its suppression of EAE (15). Furthermore, while cannabinoids suppressed EAE, this effect was dependent on the induction of IFN-β. This was evidenced by using R(+)WIN 55,212-2, a synthetic cannabinoid, whose inhibitory effect on EAE was largely reduced by an anti-IFN-β mAb (28). Consistent with these observations, we found that MAT up-regulated IFN-β production, and its role was further verified by IFN-β neutralizing antibody. The observation that mice treated with anti-IFN-β alone developed more severe clinical signs of EAE indicates an inhibitory feedback of endogenous IFN-β production against autoimmunity. More important, the suppressive effect of MAT on EAE was largely blocked by IFN-β neutralizing antibody, providing strong evidence for a pivotal role of IFN-β induction in the effect of MAT on EAE, and making it an option for inducing endogenous IFN-β production for MS patients.

IL-10 is an important anti-inflammatory cytokine that plays a crucial role in preventing various inflammatory pathologies especially in tumor and autoimmune diseases, through reducing the function of antigen-presenting cells (29, 30), and inhibiting T cell proliferation and Th1/Th17 cell differentiation. High IL-10 expression level within the CNS is considered important for the initiation of recovery from EAE, and transduction of neural stem cells with IL-10 significantly enhanced their neuroregenerative capacity (31). Consistent with our observations, IL-10 induction has also been considered a mechanism underlying the suppressive effect of MAT in several other inflammatory disorders, such as rheumatoid arthritis (32, 33), neuropathic pain (34), and nephropathy (35). These results provide evidence for the capacity of MAT to induce IL-10 production in inflammatory and autoimmune diseases, thus exerting its therapeutic effect in these diseases.

Another novel finding in our study is that MAT treatment induces IL-27 production in CD11b^+^ cells in the CNS, i.e., infiltrating macrophages/microglia. IL-27 exerts potent anti-inflammatory effects in both infection and autoimmunity (36). A range of cell types produce IL-27, including activated APCs, astrocytes and microglia (37–39). Known anti-inflammatory effects of IL-27 include suppression of IL-2 production (40) and Th17 differentiation (41–43). Specifically, IL-27 significantly inhibited both non-polarized and IL-23-driven IL-17 production by myelin-reactive T cells, thereby suppressing their encephalitogenicity in adoptive transfer EAE (37). Of importance is that IL-27 is a potent inducer of IL-10 production (44–47), and also mediates IFN-β-induced IL-10 (18, 44). Our results showing that MAT-treated EAE mice had increased levels of IFN-β, IL-27 and IL-10, and that blockade of IFN-β reduced MAT-induced IL-27 and IL-10 production, indicate an IFN-β/IL-27/IL-10 pathway as an important mechanism underlying the immunotherapeutic effect of MAT on EAE.

In summary, our study demonstrates for the first time that MAT exerts its therapeutic effect in EAE through an IFN-β/IL-27/IL-10 pathway. MAT treatment could thus be a novel, safe, low-cost, and effective therapy as an alternative to exogenous IFN-β for MS.

## Data Availability Statement

The datasets used in this manuscript are available from the corresponding author on reasonable request.

## Ethics Statement

The animal study was reviewed and approved by Bioethics Committee of Zhengzhou University.

## Author Contributions

Y-JC, W-DM, and LZ conceived and designed the experiments. Y-JC, W-DM, J-DP, and RT performed the experiments. Y-JC, W-DM, M-RW, and LZ analyzed the data. M-LZ, F-ZL, and LZ contributed to the reagents, materials, and analysis tools. Y-JC, W-DM, RT, GZ, and LZ wrote the manuscript. All authors reviewed and approved the final manuscript.

## Conflict of Interest

The authors declare that the research was conducted in the absence of any commercial or financial relationships that could be construed as a potential conflict of interest.
